# Are fishing communities another most-at-risk-population? Results of a community-based study along Lake Victoria, Uganda

**DOI:** 10.1186/1742-4690-9-S2-P216

**Published:** 2012-09-13

**Authors:** A Ssetaala, J Mpendo, A Nanvubya, S Sigirenda, L Nielsen, N Kiwanuka

**Affiliations:** 1 UVRI-IAVI HIV Vaccine Program, Entebbe, Uganda; 2 International AIDS Vaccine Initiative(IAVI), New York, USA; 3 Makerere University College of Health Sciences School of Public Health, Kampala, Uganda

## Background

A recent study reported HIV prevalence of 28.8% among high risk persons in fishing communities (FC) of Uganda, indicating that FC may be another most-at-risk-population (MARP). However, these findings do not reflect the population-based HIV prevalence in FC. We conducted a community-based study to determine the population representative HIV prevalence and incidence among FC along Lake Victoria shores, Uganda.

## Methods

Community-wide mapping and census of households in 8 fishing communities was conducted. A computer-based random sample of 2200 participants aged 18-49 years was selected for interviewing by same-sex interviewers using a semi-structured questionnaire. Blood was collected for HIV serology using rapid HIV tests as per the national algorithm.

## Results

We interviewed 2,192 (99.6%) participants, of whom fifty percent were females, median age 29 years, IQR 24-35 years and 82% had stayed in the communities for at least a year.

HIV prevalence was 26.7%, higher among females than males [32.6% vs. 20.8%, p<0.01,OR=0.5], those who were single [34.7% vs. 27.8%, p<0.01, OR=1.4], those with >5 lifetime sexual partners [32% vs. 21%, p<0.01,OR=1.7], with no formal education [39.2% vs. 25.5%, p<0.01,.OR=0.5], reported alcohol use in previous 3 months [32% vs. 20.8%, p<0.01, OR=1.8], alcohol use before sex [34% vs. 21%, p<0.01, OR=0.5] and used illicit drugs [33% vs. 25.6%, p<0.01, OR=0.7].

HIV prevalence varied by occupation, highest among sex workers (66.7%), boat makers (50%), government employees (43%), bar owners (37.6%) and bar attendants (36.4%), [p<0.01]. Prevalence increased with age [p<0.01] and was not associated with consistent condom use [26.8% vs. 26.4%, p=0.45].

## Conclusion

FC in Uganda have a disproportionately high HIV prevalence compared to the national average of 6.7%. Our prevalence estimate was comparable to that reported among high risk FC. Prevention efforts towards reducing HIV prevalence in these communities are needed, and such populations may be considered for future HIV prevention trials.

